# The effect of sodium bicarbonate mini-tablets ingested in a carbohydrate hydrogel system on 40 km cycling time trial performance and metabolism in trained male cyclists

**DOI:** 10.1007/s00421-024-05567-3

**Published:** 2024-07-28

**Authors:** Eli Spencer Shannon, Amanda Regnier, Ben Dobson, Xiaolin Yang, S. Andy Sparks, Lars Robert Mc Naughton

**Affiliations:** 1 Nutrition, Sport and Exercise Performance Research Group, Department of Sport and Physical Activity, Edge Hill University, Ormskirk, UK; 2 China Institute of Sport and Health Science, Beijing Sport University, Beijing, China; 3 Maurten AB, Gothenburg, Sweden; 4 Research Institute for Sport and Exercise Sciences, Liverpool John Moores University, Liverpool, UK

**Keywords:** Extracellular buffering, High intensity exercise, Ergogenic aid, Alkalosis

## Abstract

**Introduction:**

Sodium bicarbonate (NaHCO_3_) ingestion has been found to be ergogenic in high-intensity exercise that ranges from 1 to 10 min; however, limited studies have investigated high-intensity exercise beyond this duration.

**Purpose:**

The present study aimed to determine the effect of NaHCO_3_ ingested using a carbohydrate hydrogel delivery system on 40 km time trial (TT) performance in trained male cyclists.

**Methods:**

Fourteen trained male cyclists ingested 0.3 g kg^−1^ BM NaHCO_3_ (Maurten AB, Sweden) to determine individualised peak alkalosis, which established time of ingestion prior to exercise. Participants completed a 40 km familiarisation TT, and two 40 km experimental TTs after ingestion of either NaHCO_3_ or placebo in a randomised, double-blind, crossover design.

**Results:**

NaHCO_3_ supplementation improved performance (mean improvement = 54.14 s ± 18.16 s; *p* = 0.002, *g* = 0.22) and increased blood buffering capacity prior to (HCO_3_^−^ mean increase = 5.6 ± 0.2 mmol L^−1^, *p* < 0.001) and throughout exercise (*f* = 84.82, *p* < 0.001, pη^2^ = 0.87) compared to placebo. There were no differences in total gastrointestinal symptoms (GIS) between conditions either pre- (NaHCO_3_, 22 AU; Placebo, 44 AU; *p* = 0.088, *r* = 0.46) or post-exercise (NaHCO_3_, 76 AU; Placebo, 63 AU; *p* = 0.606, *r* = 0.14).

**Conclusion:**

The present study suggests that ingesting NaHCO_3_ mini-tablets in a carbohydrate hydrogel can enhance 40 km TT performance in trained male cyclists, with minimal GIS. This ingestion strategy could therefore be considered by cyclists looking for a performance enhancing ergogenic aid.

## Introduction

There is considerable research evidence to support the ergogenic effects of sodium bicarbonate (NaHCO_3_) for high-intensity exercise performance across a variety of durations and distances. Traditionally, NaHCO_3_ is predominantly recommended for use in high intensity exercise up to 10 min in duration (Carr et al. 2011; McNaughton 1992; McNaughton et al. 2016). This buffering agent has been shown to enhance performance in a host of disciplines and durations, such as cross-fit athletes’ 2 km rowing performance (Martin et al. 2023), regional swimmers’ latter stages of repeated swimming sprints (Gough et al. 2023) and trained cyclists’ 4 km time trial (TT) performance (Hilton et al. 2020). Although there are several key physiological factors associated with skeletal muscle fatigue during high-intensity exercise, the most common consensus is the intra-muscular and extracellular accumulation of hydrogen (H^+^) ions (Carr et al. 2011; Requena et al. 2005). The ingestion of NaHCO_3_ increases blood bicarbonate concentration (HCO_3_^−^) which facilitates an enhanced H^+^ efflux from the intracellular space (Siegler et al. 2016). This subsequently enhances the muscle contractile capacity and glycolytic rate, which can delay the onset of fatigue and improve exercise performance (Requena et al. 2005).

The underlying mechanisms that enable performance improvements following the ingestion of NaHCO_3_ is best suited for high-intensity exercise that utilises a high proportion of energy via non-oxidative pathways (de Oliveira et al. 2021; Hilton et al. 2020; McNaughton et al. 2016). However, some studies have also shown performance enhancements following the ingestion of extracellular buffers in high-intensity exercise exceeding 10 min in duration. These buffering agents include sodium citrate (SC; Potteiger et al. 1996; Oopik et al., 2004), sodium phosphate (SP; Folland et al. 2008) and lactate supplementation (Morris et al. 2011). Despite the improved performance originated from these extracellular buffering agents, NaHCO_3_ is widely regarded as the most effective extracellular buffer (de Oliviera et al., 2021) and the most readily available. Research, therefore, needs to explore whether ingestion of NaHCO_3_ can also achieve similar performance enhancements during longer duration, high intensity exercise.

Earlier research on NaHCO_3_ on prolonged high intensity exercise by George and MacLaren (1988) observed a 17% performance improvement in time to exhaustion (TTE) when running on a treadmill at a velocity corresponding to 4 mmol L^−1^ of blood lactate following a 0.2 g kg^−1^ BM dose. This magnitude of improvement is similar to the work of McNaughton et al. (1999), where 0.3 g kg^−1^ BM NaHCO_3_ enhanced total work by 13.3% in a 1 h competitive cycling TT. Additionally, Egger et al., (2014) also observed improved TTE during cycling at 110% individual anaerobic threshold (IAT), following the ingestion of 0.3 g kg^−1^ BM of NaHCO_3_ in solution. Finally, in a more ecologically valid assessment of performance, Leach et al., (2023) studied the effects of pre-exercise NaHCO_3_ in the form of gelatin and enterically coated capsules on 16.1 km TT performance. These authors observed a 2.1% and 2.5% improvement respectively, in comparison to a placebo, using an individualised time to peak alkalosis (TTP) ingestion strategy (Miller et al. 2016). This differs from the previous research investigating NaHCO_3_ on prolonged high intensity exercise as historically studies tended to use standardised ingestion strategies of 60 min (Egger et al. 2014), 90 min (McNaughton et al. 1999), and 120 min (George and MacLaren 1988).

Despite the aforementioned research demonstrating performance enhancing effects with NaHCO_3_ ingested at standardised times, implementing an individualised ingestion strategy may further increase the ergogenic effects of NaHCO_3_ (Boegman et al. 2020). Pre-determining individualised ingestion strategies accounts for the inter-individual variation in time to peak HCO_3_^−^ (Gough et al. 2017) and blood pH (Sparks et al. 2017), potentially increasing the performance enhancing effects of NaHCO_3_ ingestion (Boegman et al. 2020). Consequently, additional research is necessary to establish the effect of NaHCO_3_ on longer duration TT performance when ingested at TTP alkalosis.

The most utilised ingestion strategies of NaHCO_3_ are via solution (McNaughton et al. 2019) and gelatin capsules (Carr et al. 2011). However, gastrointestinal symptoms (GIS) are common with these modes (Hilton et al. 2020). Consequently, researchers and athletes have begun to utilise other modes of ingestion, such as enterically coated and delayed-release capsules (Hilton et al. 2019, 2020; Leach et al. 2023) and commercially available ingestion systems (Gough and Sparks 2024). As such, a new commercially available NaHCO_3_ product (Maurten Bicarb System) claims to reduce GIS and increase extracellular blood buffering capacity through the medium of NaHCO_3_ mini-tablets embedded within a carbohydrate (CHO) hydrogel. This enables the passage of the mini-tablets through the pyloric sphincter, reducing the interaction of NaHCO_3_ with stomach acid. A recent study has shown that this novel ingestion system significantly reduces GIS and extends the duration of potentially ergogenic alkalosis in comparison to NaHCO_3_ ingested in vegetarian capsules (Gough and Sparks 2024). However, the effects of NaHCO_3_ mini-tablets within a CHO hydrogel on TT performance is currently unclear. Therefore, the aim of the present study was to determine the effects of ingesting 0.3 g kg^−1^ BM NaHCO_3_ at TTP using a CHO hydrogel system on 40 km TT performance in trained male cyclists.

## Methods

### Participants

Fourteen, trained, male cyclists (age 43 ± 15 years; height 1.74 ± 0.68 m; body mass 75 ± 9 kg; body fat 15.1 ± 4.9%, VO_2Peak_ 51.9 ± 6.4 mL kg^−1^ min^−1^, HR_max_ 185 ± 8 b min^−1^, W_Peak_ 383 ± 49 W) were recruited for this study. The sample size was determined using G*Power (v3.1.9.6, Kiel University, Germany) for within factor analysis, assuming a 0.05 error probability and 0.80 power. The analysis revealed that twelve participants would be adequate to detect changes in performance with a small effect size (0.20). The participants cycled > 3 day week^−1^ and > 180 km week^−1^ and had a minimum of two years of cycling experience. Each participant regularly competed in cycling TTs. Based on these characteristics, and in accordance with performance criteria from McKay et al., (2021), participants were classed as “trained”. Individuals with clinically diagnosed GIS-related disorders, hypertension, renal impairment, and individuals who were currently ingesting nutritional buffering supplements or were currently on any type of medication were excluded from the study. Ethical approval (ETH2223-0084) was secured from the institutional research ethics committee whilst written and verbal informed consent was obtained from each participant prior to participation. All procedures were conducted in accordance with the Declaration of Helsinki (2013).

### Experimental design

In a randomised, double-blind, crossover design, participants attended Edge Hill University’s Physiology Laboratory on five separate occasions separated by at least 48 h. The first visit measured time to peak blood alkalosis (Miller et al. 2016). Following this, the second visit measured body composition (BodPod®, Cosmed, Italy) and the participant’s VO_2Peak_. The third visit comprised of a familiarisation 40 km TT. Finally, the last two visits were allocated to two 40 km TTs, in which the participants ingested either a 0.3 g kg^−1^ BM dose of NaHCO_3_ (Maurten AB, Gothenburg, Sweden) or a taste and texture matched placebo. To determine the effectiveness of the blinding, participants were asked whether they believed they had ingested NaHCO_3_ or placebo. Dietary intake was registered in a food diary 24 h prior to the first of the trials and participants were asked to replicate this in all subsequent trials. Each participant arrived at the laboratory 2 h post-prandial, whilst each laboratory visit was arranged to occur at the same time of day (± 1 h) to limit the physiological effects of circadian variations (Reilly 1990). Finally, participants refrained from alcohol consumption and strenuous activity 24 h before each experimental trial.

### Ingestion strategy

The ingestion strategy involved the consumption of 40 g of CHO hydrogel mixed with 200 mL of water, and 0.3 g kg^−1^ BM of sodium bicarbonate mini-tablets for the NaHCO_3_ trials (Maurten Bicarb System, Maurten AB, Gothenburg, Sweden). For the placebo, the water and hydrogel mixture had 6.50 g of finely crushed non-caloric sweetener (Sula®, Sula GmbH, Metelen, Germany) added in place of the NaHCO_3_ mini-tablets, to taste and texture match it. Once the CHO hydrogel was added to the water, it was mixed and left for 5 min, in accordance with the manufacturer’s instructions, then either the NaHCO_3_ or placebo were added, and it was ingested immediately.

## Data Collection.

### Peak oxygen uptake

The determination of VO_2Peak_ was achieved by administering a ramp cycling test (Deb et al. 2018). The ramp test was conducted on an electromagnetically braked cycle ergometer (Lode Excalibur Sport, Groningen, the Netherlands), and consisted of a 5 min warm-up at 70 W at a self-selected cadence between 70 and 90 rpm, followed by an increase in workload of 30 W min^−1^ until volitional exhaustion. Breath-by-breath oxygen consumption (VO_2_), carbon dioxide (VCO_2_), minute ventilation (VE) and respiratory exchange ratio (RER) were measured continuously (Vyntus, Vyaire Medical, Illinois, USA). The determination of VO_2Peak_ was defined as the highest 30 s rolling VO_2_ average of the ramp test. This occurred at volitional exhaustion when (a) RER > 1.15 and (b) rating of perceived exertion (RPE) > 18/20 AU (Midgley et al. 2007).

### Time to peak (TTP) blood alkalosis

To determine the time to reach peak alkalosis, participants rested for 10 min before an initial blood sample was collected in a 95 μL heparin-coated glass capillary tube (Radiometer Medical Ltd, Copenhagen, Denmark). Participants then remained seated and ingested 0.3 g kg^−1^ NaHCO_3_. Post-ingestion 95 μL fingertip blood samples were taken every 10 min, up to 180 min (Gough et al. 2017), and were analysed immediately using a reliable and valid (Stadlbauer et al. 2011) blood gas analyser (Radiometer ABL800 BASIC, Copenhagen, Denmark) for blood HCO_3_^−^ and pH. Peak alkalosis was defined as the greatest blood HCO_3_^−^ concentration recorded within 180 min and subsequently determined the individualised timing of ingestion for the experimental trials. Peak HCO_3_^−^ has previously been shown to be a more reliable measure of alkalosis than peak pH (Gough et al. 2017). During this time, participants were instructed to record any GIS on a 9-item, 10-point Likert Scale, (0 = no symptom, and 10 = severe symptom) following each blood sample (Carr et al. 2011). Symptoms on the 9-item scale included nausea, flatulence, stomach cramping, belching, stomach-ache, bowel urgency, diarrhoea, vomiting, and stomach bloating.

### 40 km time trials

Following a 10 min seated rest, an initial finger-tip blood sample (blood lactate, pH, HCO_3_^−^) was collected. After which, participants ingested either 0.3 g kg^−1^ NaHCO_3_ or a placebo, in a randomised order. Participants were instructed to record any GIS on the previously described 9-item, 10-point Likert scale pre-ingestion, pre-exercise, and post-exercise. Total GIS was defined as the number of individual symptom scores (Arbitrary Units; AU) per timepoint and per condition, whereby total aggregated GIS was characterised as the accumulation of individual symptoms per condition throughout both timepoints (pre-exercise and post exercise). After ingestion, participants remained rested until their individualised TTP alkalosis timepoint, after which another resting finger-tip blood sample (blood; pH, HCO_3_^−^) was drawn. Participants then completed a 5 min self-selected warm-up before starting the 40 km TT. An additional finger-tip blood sample was drawn at the end of the warm-up. All TT’s were completed using an air braked cycle ergometer (Wattbike Pro, Nottingham, UK), which began immediately following the post warm-up blood sample and were replicated in all subsequent trials.

Performance metrics including mean power, mean cadence, mean speed, and split time were recorded at 10, 20, 30, and 40 km of the TT, whilst total completion time was recorded as soon as the TT finished. Blood lactate (Lactate Pro 2 Analyser, Arkray, Japan), average heart rate (HR; Forerunner, Garmin, Olathe, Kansas, USA), RPE (6–20 scale; Borg, 2002), blood pH, HCO_3_^−^, and electrolytes (Na^+^, K^+^, Cl^−^, Ca^2+^) were measured at 10, 20, 30, and 40 km timepoints. Furthermore, at 9–10, 19–20, and 29–30 km, respiratory responses were recorded as previously described, by means of wearing a nosepiece and placing a mouthpiece in the participant’s mouth to facilitate intermittent sample collection. The TTs took place in a temperate controlled laboratory environment (temperature, 19 ± 2 °C, humidity, 45 ± 5%; pressure, 753 ± 9 mmHg). Throughout each TT, a fan (Clarke Air, Essex, UK) was placed 1.5 m away from the ergometer, to promote evaporative cooling. Participants received real time feedback on their heart rate and distance covered during each time trial, but were blinded from power-output, time-elapsed, and speed data. No verbal encouragement was provided during the TT’s.

### Statistical analyses

Assessed variables were analysed for normality (Shapiro–Wilk Test) and homogeneity of variance (Mauchly Test), whereby violations were corrected, if necessary, via Greenhouse–Geisser adjustments. To analyse overall TT performance, a paired samples t-test was conducted, and effect sizes were calculated using Hedge’s *g.* Effect sizes were considered as: trivial (< 0.20), small (0.20–0.49), moderate (0.50–0.79) or large (> 0.80) (Cohen 1988). The assumption of normal distribution was violated for GIS, therefore, a Wilcoxon signed rank test was conducted as the non-parametric alternative. Effect sizes (*r)* for non-normally distributed data were calculated from z/n, with 0.10, 0.24, and 0.37 considered as small, medium, and large, respectively (Ivarsson et al. 2013). Additionally, a two-way repeated measures (condition x time) analysis of variance (ANOVA) was conducted using a Bonferroni correction to analyse changes in mean power, split time, mean cadence, mean speed, acid–base balance (blood HCO_3_^−^ and blood pH), electrolytes (Na^+^, K^+^, Cl^−^, Ca^2+^), RPE, physiological variables (HR, blood lactate), and respiratory responses (VE, VO_2_, VCO_2_ and RER) during the experimental trials. Effect sizes for pη^2^ were interpreted as small (0.01), medium (0.06) and large (0.14) (Cohen 1988). Data are presented as mean ± standard deviation (SD), unless stated otherwise. Data were analysed using SPSS Statistics (Version 29, IBM SPSS Inc., Chicago, IL, USA), with significance set at *p* < 0.05.

## Results

### 40 km tine trial performance

**Fig. 1:**
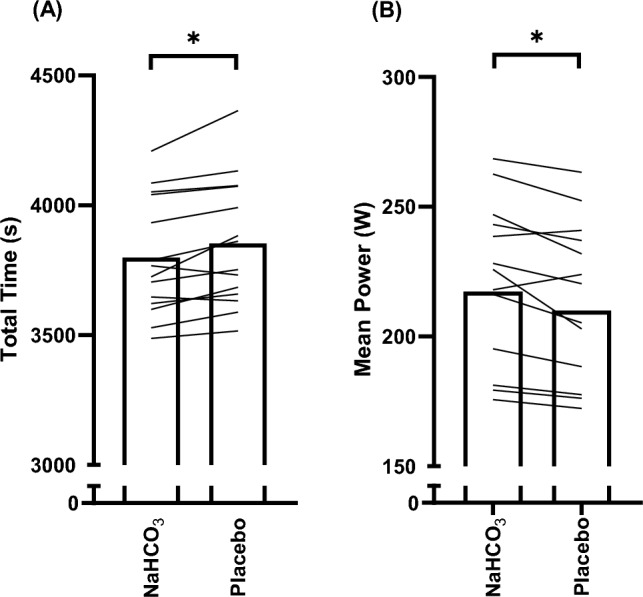
Mean and individual values for total time (**A**), and mean power (**B**) following sodium bicarbonate (NaHCO_3_) and placebo ingestion. “*” denotes a significant difference between conditions (*p* < 0.01)

**Fig. 2:**
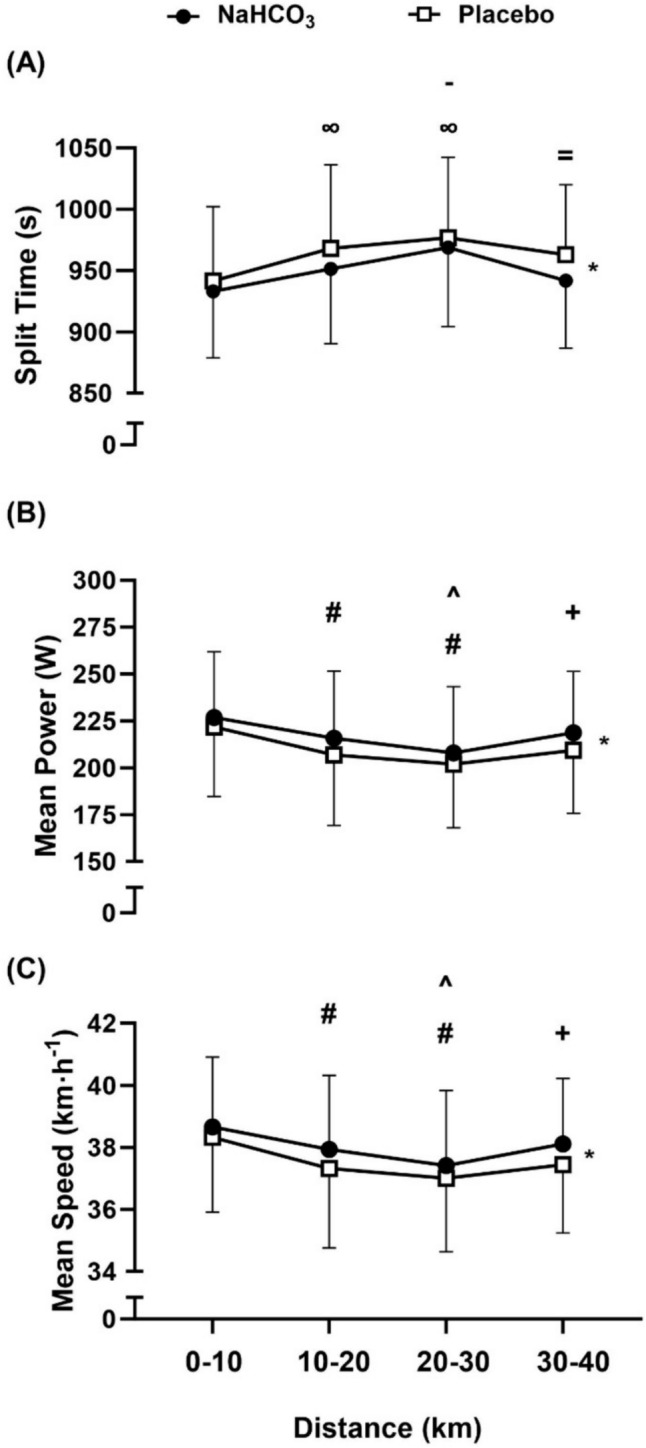
Mean ± SD split time (**A**), mean power (**B**) and mean speed (**C**). “**∞**” denotes significant increase from first timepoint (0–10 km) (*p* < 0.01), “**-**” denotes significant increase from second timepoint (10–20 km) (*p* < 0.05), “ = ” denotes significant decrease from third timepoint (20–30 km) (*p* < 0.05),** “#”** denotes significant decrease from first timepoint (0–10 km) (*p* < 0.001), “^” denotes significant decrease from second timepoint (10–20 km) (*p* < 0.01), and + denotes significant increase from third timepoint (20–30 km) (*p* < 0.05). “*” denotes a significant condition difference throughout all timepoints (*p* < 0.01)

Overall TT performance (Figs. 1 and 2) was improved (mean improvement = 54.14 ± 18.16 s) following ingestion of 0.3 g kg^−1^ BM NaHCO_3_ compared to the placebo (total time, *t* = 3.75, *p* = 0.002, *g* = 0.22; overall mean power output, *t* = 3.72, *p* = 0.003, *g* = 0.21; mean power output, *f* = 13.83, *p* = 0.003, pη^2^ = 0.516; mean speed, *f* = 14.24, *p* = 0.02, pη^2^ = 0.002; split time, *f* = 13.88, *p* = 0.003, pη^2^ = 0.52). Twelve out of the fourteen participants performed better following NaHCO_3_ supplementation in comparison to the placebo (Fig. 1). There was no significant trial order effect (*t* = 0.91, *p* = 0.38, *g* = 0.07) and familiarisation performance times were not significantly different to placebo (mean difference (MD) = -36.21 s, *p* = 0.056, *g* = 0.14), suggesting minimal variability for the 40 km TT performance test and no significant placebo effect. A little over half (57%) of the participants correctly guessed which supplement was ingested. There was no significant condition*distance interaction effect between mean power output (*f* = 2.02, pη^2^ = 0.355, *p* = 0.355), mean speed (*f* = 2.22, pη^2^ = 0.377, *p* = 0.143), or split time (*f* = 2.15, *p* = 0.14, pη^2^ = 0.14), suggesting minimal differences in pacing strategy in the TTs between both conditions.

### Acid-base balance

**Fig. 3:**
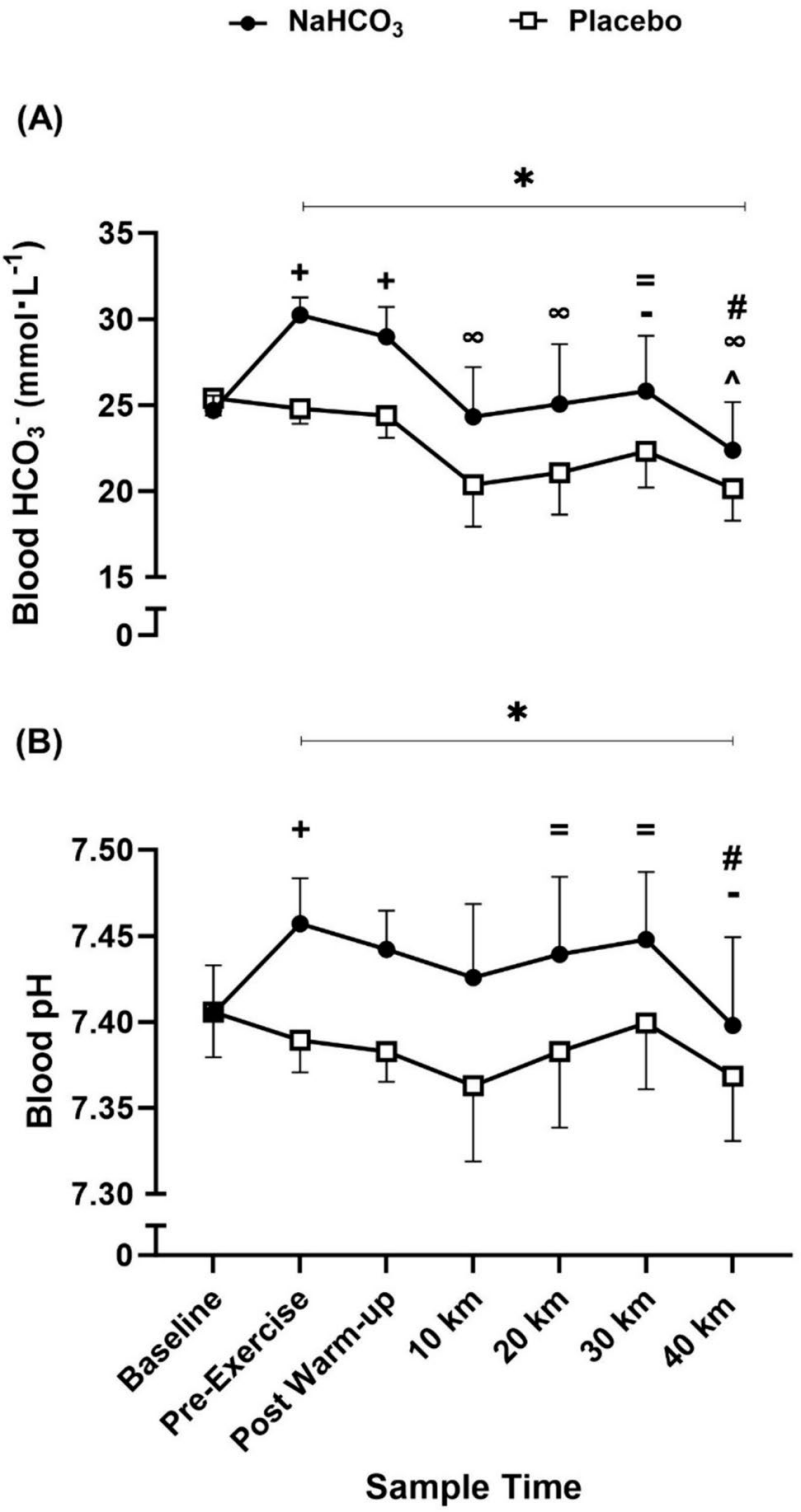
Mean ± SD blood HCO_3_^−^ (**A**) and blood pH (**B**) responses before and throughout 40 km TT after NaHCO_3_ and placebo ingestion. “** + **” denotes significant increase from first time point (baseline) (*p* < 0.01), “**∞**” denotes significant decrease from first (pre-exercise) and second (post warm-up) time point (*p* < 0.01), “**-**” denotes significant decrease from second time point (pre-exercise) (*p* = 0.01), “** = **” denotes significant increase from fourth time point (10 km) (*p* < 0.05), “**#**” denotes significant decrease from sixth time point (30 km) (*p* < 0.005), “*” denotes a significant condition difference between NaHCO_3_ and placebo conditions (*p* < 0.01)

Blood acid–base balance (blood HCO_3_^−^ and blood pH) at baseline was similar between conditions (blood HCO_3_^−^, MD = 0.34 mmol L^−1^, *p* = 0.082; blood pH, MD = 0.002 pH units, *p* = 0.83). The ingestion of NaHCO_3_ significantly increased blood HCO_3_^−^ and blood pH in comparison to the placebo (*f* = 84.82, *p* < 0.001, pη^2^ = 0.87, *f* = 91.04, *p* < 0.001, pη^2^ = 0.88, respectively). Both of these blood variables increased beyond baseline measurements following ingestion of NaHCO_3_ at pre-exercise (blood HCO_3_^−^, MD = 5.6 mmol L^−1^, *p* < 0.001; blood pH, MD = 0.052 pH units, *p* = 0.001) and remained elevated at post warm-up (blood HCO_3_^−^, MD = 4.27 mmol L^−1^, *p* < 0.001; blood pH, MD = 0.037 pH units, *p* = 0.01). From baseline measurements to the end of the 40 km TT, blood HCO_3_^−^ and blood pH changed considerably (*f* = 28.28, *p* < 0.001, pη^2^ = 0.69, *f* = 5.84, *p* < 0.001, pη^2^ = 0.31, respectively). Between NaHCO_3_ and placebo conditions, both blood metabolites followed comparable patterns during each 40 km TT, each increasing from 10 to 30 km, followed by a subsequent decrease from 30 to 40 km (Fig. 3). This resulted in significant condition-time interaction effects for blood HCO_3_^−^ (*f* = 22.74, *p* < 0.001, pη^2^ = 0.64) and blood pH (*f* = 12.10, *p* < 0.001, pη^2^ = 0.48).

### Respiratory responses

**Fig. 4:**
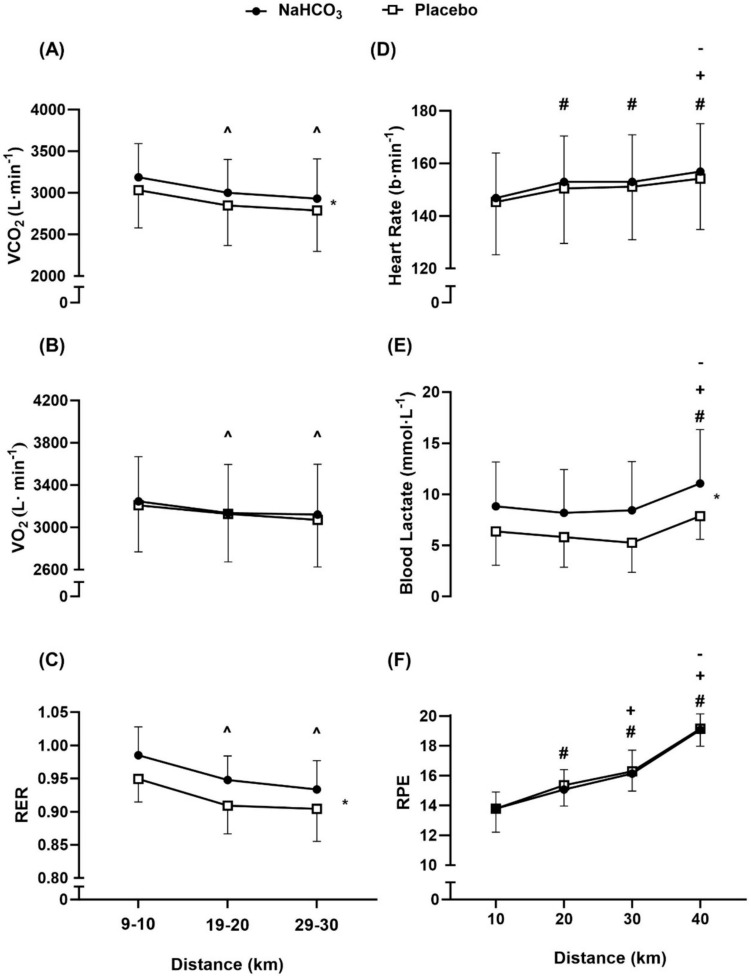
Mean ± SD VCO_2_ (**A**), VO_2_ (**B**), RER (**C**), heart rate (**D**), blood lactate (**E**), and RPE (**F**) responses throughout 40 km TT after NaHCO_3_ and placebo ingestion. “**^**” denotes significant decrease from first timepoint (9-10 km) (*p* < 0.05). “**#**” denotes significant increase from first timepoint (10 km) (*p* < 0.001). “**+**” denotes significant increase from second timepoint (20 km) (*p* < 0.01). “**-**” denotes significant increase from third timepoint (30 km) (*p* < 0.01). “*” denotes significant condition difference at all timepoints (*p* < 0.01)

The VO_2_ responses (Fig. 4) did not differ between conditions (*f* = 0.42, *p* = 0.52, pη^2^ = 0.031), but VCO_2_ was higher following ingestion of NaHCO_3_ (*f* = 11.41, *p* = 0.005, pη^2^ = 0.47). Consequently, RER was also increased in the NaHCO_3_ TT’s (*f* = 12.92, *p* = 0.003, pη^2^ = 0.50). Additionally, VO_2_, VCO_2_, and RER followed similar patterns during each TT (VO_2,_ *f* = 7.96, *p* = 0.006, pη^*2*^ = 0.38; VCO_2_, *f* = 16.13, *p* < 0.001, pη^*2*^ = 0.73; RER, *f* = 47.33, *p* < 0.001, pη^*2*^ = 0.50), each decreasing after the opening 9–10 km. Minute ventilation (VE) was unaffected by either method of treatment (condition, *f* = 0.14, *p* = 0.71, pη^*2*^ = 0.011; distance, *f* = 3.61, *p* = 0.059, pη^*2*^ = 0.38). There was no condition*distance interaction effect observed for either of the respiratory response measurements (VO_2_, *f* = 0.31, *p* = 0.73, pη^*2*^ = 0.024; VCO_2_, *f* = 0.17, *p* = 0.98, pη^*2*^ = 0.003; RER, *f* = 0.59, *p* = 0.57, pη^*2*^ = 0.089).

### Heart rate, blood lactate, RPE, and cadence

Despite the present study revealing a performance difference, both heart rate and RPE were similar across both TTs (Fig. 4; heart rate, *f* = 0.98, *p* = 0.34, pη^*2*^ = 0.070; RPE, *f* = 0.59, *p* = 0.46, pη^*2*^ = 0.043). Additionally, cadence was unaffected by either condition (*f* = 0.99, *p* = 0.35, pη^2^ = 0.069) or distance (*f* = 0.345, *p* = 0.168, pη^*2*^ = 0.35). Conversely, blood lactate was higher throughout each 10 km split in the NaHCO_3_ trial compared to the placebo (*f* = 18.35, *p* < 0.001, pη^*2*^ = 0.59,). Heart rate and RPE each increased over the course of the TT (heart rate, *f* = 24.27, *p* < 0.001, pη^*2*^ = 0.87; RPE, *f* = 104.17, *p* < 0.001, pη^*2*^ = 0.97), whereas blood lactate maintained a similar pattern over the course of the TT, with responses being greater at 40 km in comparison to every other timepoint (*f* = 6.98, *p* = 0.005, pη^*2*^ = 0.35). This subsequently led to no condition*distance interaction effect between either of these measures (cadence, *f* = 0.31, *p* = 0.82, pη^2^ = 0.078; heart rate, *f* = 0.98, *p* = 0.34, pη^*2*^ = 0.070; RPE, *f* = 0.51, *p* = 0.68, pη^*2*^ = 0.122; blood lactate, *f* = 0.25, *p* = 0.89*,* pη^*2*^ = 0.65).

### Electrolyte analysis

Blood [Na^+^] following NaHCO_3_ supplementation was increased throughout the TTs (Table 1; *f* = 112.75, *p* < 0.001, pη^*2*^ = 0.90). In contrast, throughout the NaHCO_3_ TTs, blood [K^+^] was reduced in comparison to the placebo TTs (*f* = 17.87*, p* < 0.001, pη^*2*^ = 0.58). Likewise, following NaHCO_3_ supplementation, both blood [Ca^2+^] (*f* = 109.69, *p* < 0.001, pη^*2*^ = 0.89, and blood [Cl^−^] (*f* = 18.43 *p* < 0.001, pη^*2*^ = 0.59) decreased in comparison to the placebo TTs. Only blood [Ca^2+^] followed a similar pattern during the TTs throughout both conditions (*f* = 3.09, *p* < 0.001, pη^*2*^ = 0.19). There was no condition*distance interaction effect for [K^+^] (*f* = 1.59, *p* = 0.25, pη^*2*^ = 0.30), [Ca^2+^] (*f* = 0.52, *p* = 0.68, pη^*2*^ = 0.04), [Cl^−^] (*f* = 0.50, *p* = 0.69, pη^*2*^ = 0.57), or [Na^+^] (*f* = 1.46, *p* = 0.69, pη^*2*^ = 0.29) (Table 1).

**Table 1:** Mean (±SD) electrolyte responses at each quartile interval during the TT’s

Electrolyte	Condition	Distance (km)			
		10	20	30	40
K^+^	NaHCO_3_ Placebo	5.55 ± 0.27^*^ 5.81 ± 0.52	5.60 ± 0.46^*^ 6.23 ± 0.75	5.65 ± 0.60^*^ 6.17 ± 0.88	5.71 ± 0.62^*^ 5.79 ± 0.71
Na^+^	NaHCO_3_ Placebo	146.5 ± 2.1^*^ 142.2 ± 2.6	146.6 ± 2.4^*#^ 143.4 ± 2.9	147.2 ± 2.5^*#^ 143.4 ± 2.8	147.2 ± 2.9^*^ 142.6 ± 2.7
Ca^2+^	NaHCO_3_ Placebo	1.17 ± 0.02^*^ 1.24 ± 0.03	1.16 ± 0.02^*^ 1.24 ± 0.03	1.15 ± 0.02^*+**∞**^ 1.23 ± 0.02	1.17 ± 0.04^*^^ 1.24 ± 0.03
Cl^−^	NaHCO_3_ Placebo	105.9 ± 1.5^*^ 107.4 ± 2.2	106.2 ± 2.2^*^ 108.6 ± 2.9	106.0 ± 1.9^*^ 107.9 ± 2.4	105.6 ± 2.1^*^ 107.4 ± 3.0

“#” denotes significant increase from 10 km (*p* < 0.05). “** + **” denotes significant decrease from 10 km (*p* < 0.001). “**∞**” denotes significant decrease from 20 km (*p* < 0.05). “**^**” denotes significant increase from 30 km (*p* < 0.05). “*” denotes significant condition difference at all timepoints (*p* < 0.001)

### Gastrointestinal responses

Prior to exercise in both experimental conditions, no participant experienced any GIS. Additionally, the present study revealed no significant differences between either condition for total GIS after ingestion and prior to exercise (NaHCO_3_, 22 AU; Placebo, 44 AU; *z* = − 1.71, *p* = 0.088, *r* = 0.46), post exercise (NaHCO_3_, 76 AU; Placebo, 63 AU; *z* =− 0.51, *p* = 0.61 *r* = 0.14) or for total aggregated GIS (NaHCO_3_, 98 AU; Placebo, 107 AU; *z* = − 1.45, *p* = 0.15, *r* = 0.39). Peak GIS was also not influenced at either stage (Table 2; pre-exercise, z = − 1.47, *p* = 0.14, *r* = 0.39; post-exercise, z = − 0.04, *p* = 0.97, *r* = 0.011).

**Table 2:** Most severe gastrointestinal symptom (GIS) experienced by each participant measured by peak severity score (arbitrary units on 0–10 scale; AU) at pre- and post- exercise following ingestion of NaHCO_3_ or placebo

Participant	NaHCO_3_		Placebo	
	Pre-Exercise	Post-Exercise	Pre-exercise	Post-Exercise
1	Stomach cramping (1)	Flatulence (1)	Nil	Flatulence (1)
2	Stomach cramping (2)	Nausea (2)	Stomach cramping (1), stomach-ache (1)	Nausea (5)
3	Stomach bloating (2)	Nausea, flatulence (4)	Flatulence (2), stomach-ache (2)	Nausea (3), Flatulence (3)
4	Stomach-ache (5)	Nausea (5)	Flatulence (1)	Nausea (3)
5	Belching (3)	Belching (2)	Belching (3)	Nil
6	Nil	Nil	Nil	Stomach cramping (2)
7	Nil	Nil	Flatulence (1), stomach cramping (1)	Flatulence (3)
8	Nil	Nil	Nil	Nausea (1), flatulence (1)
9	Stomach-ache (2)	Stomach bloating (2)	Stomach-ache (2)	Stomach bloating (1)
10	Belching (3)	Stomach cramping (6)	Belching (2)	Stomach-ache (2), stomach cramping (2)
11	Nil	Nil	Nil	Flatulence (1)
12	Nil	Nil	Stomach cramping (1)	Nil
13	Diarrhoea (1), belching (1)	Flatulence (2)	Stomach cramping (1)	Flatulence (1)
14	Nil	Nil	Stomach cramping (1), stomach-ache (1)	Flatulence (1)

## Discussion

The primary aim of the present study was to investigate the effects of NaHCO_3_ ingestion on 40 km TT cycling performance in trained male cyclists. This is the first study to observe, that ingestion of 0.3 g kg^−1^ of NaHCO_3_ supplementation in the form of mini-tablets in a CHO hydrogel (Maurten Bicarb System) improves 40 km TT cycling performance by 1.42%. This represents a small, but practically important performance change that is larger than the variability for TT performances in trained cyclists (Laursen et al. 2003; Palmer et al. 1996; Smith et al. 2001), which has been established as being less than 1% (Currell and Jeukendrup 2008). In addition to these ergogenic effects, the present study reported no differences in GIS, HR or VO_2_ between the NaHCO_3_ ingestion and that observed in the placebo trials, despite increased performance in the NaHCO_3_ trial. Given the widespread evidence and research showing that NaHCO_3_ is well-known for producing GIS through traditional ingestion methods (Hilton et al. 2019), these findings suggest that this novel form of ingestion can serve as a practical ergogenic aid to enhance prolonged high-intensity exercise performance in trained male cyclists.

Currently, less research has investigated NaHCO_3_ supplementation on prolonged high-intensity exercise performance that extends beyond 10 min in duration. Despite this, in the few studies that have used NaHCO_3_ ingestion for prolonged high-intensity TT exercise performance, together with the present findings, its ergogenic potential for TT’s appears to extend beyond the perception that it is only useful for short duration exercise. George and MacLaren (1988) found a 17% improvement in TTE with a mean improvement of 263 s, whereas McNaughton et al. (1999) saw total work increase by 13.3% during a 60 min effort. This too, is similar to the improvements in total work at 110% IAT observed by Egger et al., (2014) following NaHCO_3_ supplementation. In contrast, despite a significant increase of metabolic alkalosis by means of increases in blood pH and HCO_3_^−^, cycling for ~ 30 min at 80% VO_2peak_ (Stephens et al. 2002) and running at 110% IAT until exhaustion (Freis et al. 2017), were previously shown to be unaffected by NaHCO_3_ supplementation. The lack of performance improving effects following NaHCO_3_ supplementation on prolonged high intensity exercise in these studies could be attributed to the adverse GIS experienced in participants (Freis et al. 2017), or due to exercise performance protocols with poorer reliability. Indeed, most of the work on NaHCO_3_ and prolonged high intensity exercise has commonly utilised TTE as a measurement of performance along with either NaHCO_3_ ingestion in fluid or gelatin capsules. However, measurement by means of TTs have been found to have a lower coefficient of variation (CV) in comparison to TTE, which is observed as improved reproducibility (Palmer et al. 1996). This may in part explain why the present study and Leach et al., (2023) were able to observe improvements in more prolonged cycling TT performance following the ingestion of NaHCO_3_ than in many other previous studies.

The improvement in 40 km TT performance in the present study was observed following the ingestion of NaHCO_3_ at an individualised timepoint, coinciding the start of exercise with peak alkalosis (Miller et al. 2016). There are comparatively fewer studies that have implemented individualised NaHCO_3_ ingestion strategies exploring high-intensity exercise longer than 10 min (Lassen et al. 2021; Leach et al. 2023). Interestingly, work by Carr et al. (2011) suggests, to evoke an ergogenic response following NaHCO_3_ supplementation, a 5 or 6 mmol L^−1^ increase in blood HCO_3_^−^ is necessary. This, however, differs from the work of Leach et al. (2023), whereby enterically coated NaHCO_3_ improved 16.1 km TT performance by 2.5%, despite only producing a mean 3.7 mmol L^−1^ increase in blood HCO_3_^−^, but such ingestion strategies have previously been shown to have lower peak HCO_3_^−^ responses than traditional methods of ingestion (Hilton et al. 2019). Additionally, Gough et al. (2022) reported an increase of 2.8 mmol L^−1^ in blood HCO_3_^−^ compared to a placebo, which enhanced 4 km cycling TT performance by 1.6% in hot (30 °C) conditions. Both studies are below the previously acclaimed theoretical metabolic threshold to enhance performance. Interestingly, the mean increase in HCO_3_^−^ that Leach et al. (2023) observed in gelatin encapsulation (5.7 mmol L^−1^) is similar to the present study’s pre-exercise HCO_3_^−^ of 5.6 mmol L^−1^. What is clear, is that the acid–base response between ingestion methods is different. The present study optimised ingestion time, observed a large change in blood HCO_3_^−^, and found little or no GIS. These factors may therefore maximise the potential of observing performance improvements but given the likely HCO_3_^−^ responses following NaHCO_3_ using this ingestion method (Gough and Sparks 2024), it is not clear if an individualised ingestion time is required. Further research is therefore required to ascertain the effectiveness of NaHCO_3_ on ecologically valid, high-intensity exercise performance tasks, using a wide variety of ingestion types and timing methods.

In the current study, blood metabolites, such as HCO_3_^−^, pH, and lactate were elevated to a greater extent throughout the TT following NaHCO_3_ supplementation. Following NaHCO_3_ supplementation, during prolonged high-intensity exercise, a higher efflux of lactate has been observed (Hollidge-Horvat et al. 2000; Siegler et al. 2016; Egger et al. 2014). Work by Hollidge-Horvat et al. (2000) suggests that exercise-induced metabolic alkalosis may increase the rate of glycolysis, resulting in a difference in pyruvate production leading to elevated lactate concentrations. Increased blood lactate concentrations have also been shown to result from improved lactate efflux following alkalosis, even without alterations to skeletal muscle function (Spriet et al. 1986). Intriguingly, improved performance times and power output occurred in the present study, without changes to cadence, heart rate, RPE, or VO_2_, suggesting increased torque and efficiency, which was not previously observed in 2 km TT performance (Voskamp et al. 2020). The observed increases in VCO_2_ throughout the 40 km TT following NaHCO_3_ supplementation may also suggest increased glycolytic rate, increased H^+^ efflux and buffering (Hirakoba et al. 1993; Requena et al. 2005), or both given the differences in power output. This is however contradictory to previous meta-analytical data deeming NaHCO_3_ inadequate to enhance VCO_2_ production throughout aerobic exercise (Calvo et al. 2021), but this may be explained by the diverse study methods, participants and ingestion protocols included in their analysis. Nonetheless RER was elevated in present study, an observation which was also made by Egger et al., (2014). Clearly, further research is required to investigate these responses following the use of the present NaHCO_3_ ingestion strategy.

The current study also observed decreases in [K^+^] throughout the TT following NaHCO_3_ supplementation. Previous research has suggested a potential association between the reduction of H^+^ in the interstitium following induced metabolic alkalosis with a subsequent decrease in [K^+^]. This may provide a potential protective function against the reduction in muscle force and fatigue, induced by [K^+^] (Douroudos et al. 2006). The increased extracellular buffering capacity explained by increases in HCO_3_^−^ and blood pH, in addition to the increases in blood lactate, and decreases in [K^+^], likely further explain the improvements in 40 km TT performance derived by NaHCO_3_ supplementation and warrant further investigation in prolonged exercise.

Ingestion of NaHCO_3_ is renowned for causing GIS (Carr et al. 2011; Kahle et al. 2013; McNaughton et al. 2019), whereby previous NaHCO_3_ research has observed ergolytic effects (Cameron et al. 2010), with some research only reporting significant performance improvements when participants with GIS were excluded from the statistical analyses (Saunders et al. 2014). The results of the present study align with recent research suggesting that the delivery method of mini-tablets in CHO hydrogel reduces GIS in comparison to traditional delivery methods of GIS, such as encapsulation (Gough and Sparks 2024). In the present study, six of the participants reported no GIS after NaHCO_3_ supplementation and those that did, reported only minor to mild GIS responses. Therefore reducing, or in some cases, eliminating, the GIS response has the potential to enhance the ergogenic capacity of this ergogenic aid. Interestingly, all participants reported some GIS in the placebo trial, but this may have been due to the presence of isomalt, and the performance of high intensity prolonged cycling. Further work is therefore required to compare the GIS responses following ingestion of the Maurten Bicarb System to other placebos.

## Future directions and limitations

The present study had limitations that should be acknowledged. The results are specific to trained male cyclists, so using the present data to make inferences to other populations should be done with caution. Importantly, sex-based differences are possible (Carr et al. 2023), given that female biological differences such as lower muscle mass and number of type II muscle fibres, may lead to decreased whole body glycolytic rate, potentially resulting in lower H^+^ accumulation (Hegge et al. 2016; Janssen et al. 2000; Russ et al. 2005). The inclusion of an additional control and NaHCO_3_ trial would have been useful to specifically determine the test–retest reliability in the present participant population and to verify the performance improvements. However, researchers must clearly balance what might be ideal, with the practicalities of actually being able to recruit enough participants and being able to have them complete all trials in a short enough period that training status changes do not add an additional variable into the study. Consequently, based on experience of running many similar studies, the present design was chosen.

The observed respiratory responses were unexpected, consequently, gas samples were only obtained at three time points during in the TT’s. Future work should therefore attempt to shed more light on the potential impact of NaHCO_3_ on efficiency during exercise or by specifically conducting economy assessment protocols. The present study only investigated one dose of NaHCO_3_, similar to that recommended by the manufacturer. However, it is not clear if different doses of NaHCO_3_ are as or more effective than 0.3 g kg^−1^ using this delivery system. With such a method of ingestion that limits GIS, future research should therefore consider a dose response study design. Future research should also investigate the use of this NaHCO_3_ ingestion strategy in participants with a different training status and in females.

## Conclusion

The present study is the first to investigate 40 km TT cycling performance following an individualised ingestion protocol of NaHCO_3_ in the form of mini-tablets in a CHO hydrogel. The study demonstrates that ingestion of 0.3 g kg^−1^ NaHCO_3_ in this form can enhance 40 km TT performance by 1.42% and causes minimal GIS in a trained male cyclist population. The performance enhancement in 40 km TT cycling performance following NaHCO_3_ ingestion is likely due to an increased blood buffering capacity, with reduced relative oxygen cost suggesting improved gross efficiency. The present study demonstrates a large blood HCO_3_^−^ response with low GIS, which was similar to the GIS of a low sodium containing placebo, can improve 40 km TT cycling performance. Athletes, sports nutritionists, and practitioners should therefore consider the potential for this method of NaHCO_3_ ingestion to be ergogenic for prolonged high-intensity exercise.

## Data Availability

The data that support the findings of this study are available from the corresponding author, ESS, upon request.

## Abbreviations

ANOVA: Analysis of Variance
Ca^2+^: Calcium
CHO: Carbohydrate
Cl^−^: Chloride
CO_2_: Carbon dioxide production
CV: Coefficient of variation
GIS: Gastrointestinal symptoms
H^+^: Hydrogen ion
HCO_3_^−^: Bicarbonate anion
IAT: Individual anaerobic threshold
K^+^: Potassium
Na^+^: Sodium
NaHCO_3_: Sodium bicarbonate
RER: Respiratory exchange ratio
RPE: Rating of perceived exertion
TT: Time trial
TTE: Time to exhaustion
TTP: Time to peak
VO_2_: Oxygen uptake
VO_2Peak_: Peak oxygen uptake
